# Correction to: Elevated plasma IL-6 and CRP levels are associated with adverse clinical outcomes and death in critically ill SARS-CoV-2 patients: infammatory response of SARS-CoV-2 patients

**DOI:** 10.1186/s13613-021-00879-5

**Published:** 2021-06-09

**Authors:** Jean-Rémi Lavillegrand, Marc Garnier, Agathe Spaeth, Nathalie Mario, Geofroy Hariri, Antoine Pilon, Enora Berti, Fabienne Fieux, Sara Thietart, Tomas Urbina, Matthieu Turpin, Lucie Darrivere, Muriel Fartoukh, Franck Verdonk, Guillaume Dumas, Alain Tedgui, Bertrand Guidet, Eric Maury, Yannick Chantran, Guillaume Voiriot, Hafd Ait-Oufella

**Affiliations:** 1 Service de Médecine Intensive-Réanimation, Hôpital Saint-Antoine, Assistance Publique-Hôpitaux de Paris, 184 rue du faubourg Saint-Antoine, 75571 Paris cedex 12, France; 2 Sorbonne Université, Paris, France; 3 Service D’Anesthésie-Réanimation, Hôpital Saint-Antoine, Assistance PubliqueHôpitaux de Paris, Paris, France; 4 Département de Biochimie, Hormonologie et Suivi Thérapeutique, Hôpital Saint-Antoine, Assistance Publique-Hôpitaux de Paris, Paris, France; 5 Service de Médecine Intensive-Réanimation, Hôpital Tenon, Assistance Publique-Hôpitaux de Paris, Paris, France; 6 Inserm U970, Cardiovascular Research Center, Université de Paris, Paris, France; 7 Départe- Ment D’Immunologie Biologique, Hôpital Saint-Antoine, Assistance PubliqueHôpitaux de Paris, Paris, France

## Correction to: Ann Intensive Care 11:9 (2021) https://doi.org/10.1186/s13613-020-00798-x

After publication of the original article [1], an error was identified in Lucie Darrivere’s name.

The incorrect name is: Lucie Darrivière.

The correct name i: Lucie Darrivere.

The original article has been corrected.
